# A Probable Way Vitamin D Affects Autism Spectrum Disorder: The Nitric Oxide Signaling Pathway

**DOI:** 10.3389/fpsyt.2022.908895

**Published:** 2022-05-26

**Authors:** Bing Wang, HanYu Dong, HongHua Li, XiaoJing Yue, Lin Xie

**Affiliations:** ^1^Department of Nutrition and Food Hygiene, School of Public Health, Jilin University, Changchun, China; ^2^Department of Developmental and Behavioral Pediatrics, First Affiliated Hospital of Jilin University, Changchun, China

**Keywords:** autism spectrum disorder, nitric oxide, vitamin D, nitric oxide synthase, arginine

## Abstract

Vitamin D (VD) deficiency during pregnancy and early brain development is an important environmental risk factor for autism spectrum disorder (ASD). Its specific mechanism of action is still unclear. However, one study on the correlation between metabolomics and VD levels in children with ASD has found that the whole-blood arginine (Arg) levels of children with ASD are significantly negatively correlated with serum VD levels, suggesting that the effect of VD on ASD may be related to the signaling pathway involving Arg. Arg is a precursor of nitric oxide (NO), and changes in its levels most directly affect NO levels and signal transduction pathways. NO, a biologically active free radical, is both a neurotransmitter and a neuromodulator in the central nervous system and is related to the pathogeneses of various neurological diseases. The NO signaling pathway is not only affected by VD levels but also closely related to ASD through a series of mechanisms, such as neurotransmitter imbalance, immune disorders, and oxidative stress. Therefore, the effect of VD on ASD may be achieved via regulation of the NO signaling pathway. The current review discusses the relationship among VD, NO, and ASD as suggested by a large body of evidence in the literature in an effort to provide clues for researchers on the pathogenesis of ASD and the mechanism of VD's impact on ASD.

## Introduction

Autism spectrum disorder (ASD) refers to a group of neurodevelopmental disorders that start in early childhood. These disorders, whose main symptoms are social communication impairment and repetitive stereotyped behaviors and interests, have a high prevalence and high disability rate. ASD is one of the fastest-growing severe diseases in the world (1). In 2016, the World Health Organization estimated that 1 child among every 160 has ASD worldwide. In 2018, the US Centers for Disease Control and Prevention reported that the prevalence of ASD in 8-year-old children was as high as 1/59 (2); in 2020, the prevalence reached 1/54 and still maintained a large male-to-female ratio (4.3:1) (3). The onset of the disease occurs during childhood, and the disease often persists throughout life. Its etiology and pathogenesis are still unclear, so it remains one of the most challenging global health problems. It is generally believed that ASD is the result of the combined effects of genetic factors and environmental factors (4, 5).

Nutrition is an environmental factor that interacts with genes from the beginning of the embryonic period and has major impacts on organisms. Vitamin D (VD) is an important nutrient associated with ASD. In 2008, Cannell's epidemiological survey found that the levels of VD in the American population decreased year over year, while the prevalence of ASD increased year over year during the same period; in addition, the prevalence was high in areas with heavy air pollution, cities, and high-altitude areas where VD absorption is reduced. This evidence, combined with evidence of the effects of VD on brain development, led to the hypothesis that VD deficiency may be the etiological factor leading to ASD (6). Since then, research on the correlation between VD and ASD has received extensive attention in academic circles. Subsequent studies have shown that maternal VD deficiency during pregnancy and early brain development is an important environmental risk factor for the development of ASD. However, its specific mechanism of action remains unclear. In a study on the correlation between metabolomics and VD levels in children with ASD, our research team found that the levels of whole-blood arginine (Arg) in children with ASD are significantly negatively correlated with the levels of serum VD, suggesting that the effect of VD on ASD may be related to signaling pathways involving Arg (7). Arg is the precursor of nitric oxide (NO), which most directly affects the NO signal transduction pathway. NO, a biologically active free radical, is both a neurotransmitter and a neuromodulator in the central nervous system and is related to the pathogeneses of various neurological diseases (8). According to both domestic and international studies, the NO signaling pathway is not only affected by VD levels but also closely related to ASD through a series of mechanisms, such as neurotransmitter imbalance, immune disorders, and oxidative stress. Therefore, the effect of VD on ASD may be achieved via regulation of the NO signaling pathway. The current review discusses the relationship among VD, NO and ASD as suggested by a large body of evidence in the literature in an effort to provide clues for researchers on the pathogenesis of ASD and the mechanism of VD's impact on ASD.

## VD and ASD

VD is a fat-soluble vitamin and is the only chemical substance in our body that is obtained from sunlight. It is also known as the “sunshine vitamin.” VD is obtained in very small amounts from a normal diet, but it is primarily synthesized endogenously in the skin after irradiation by ultraviolet light. After being synthesized, VD binds to plasma VD-binding protein and is transported to the liver, where it is catalyzed by 25-hydroxylase to generate 25(OH)D; then, 25(OH)D is further hydroxylated by 1α-hydroxylase in the kidneys to generate the biologically active compound 1,25(OH)_2_D. 1, 25(OH) _2_D can also activate a variety of target genes through the VD receptor (VDR) to affect the activities of most organs in the body, including the brain. 25(OH)D is the main form of VD in the blood circulation and is used as an indicator of the nutritional VD status.

It was previously believed that the main role of VD was to regulate calcium and phosphorus metabolism and affect bone growth and development. In recent years, however, studies have shown that changes in VD levels can increase the risks of breast cancer, diabetes, dyslipidemia, cardiovascular disease, etc. (9–11). With the discovery that large amounts of 1α hydroxylase (the key enzyme in 25(OH)D synthesis) and VDR are widely present in the brain, it has been gradually recognized that VD plays important roles in brain activity (12). For example, VD plays a very important role in brain development (13, 14). VD deficiency in early life can lead to abnormal brain development and can impair learning, memory, cognition and other functions (15, 16). VD deficiency is also closely related to depression, seasonal psychosis, schizophrenia, Alzheimer's disease and other diseases (17–21). These findings suggest that this “sunshine vitamin” is a valuable sun-derived resource for humans and that its deficiency at different stages of human development and under different genetic backgrounds may have significant impacts on human health.

### Studies Have Shown That Low VD Levels Are Closely Related to ASD

In 2010, Meguid et al. reported that the serum 25(OH)D levels of Egyptian children with ASD were lower than those of normal control children (22). Subsequently, decreased VD levels in children and adolescents with ASD have been reported around the world (23–28) and found to be correlated with the clinical symptoms of ASD (29–34). Maternal vitamin D levels during pregnancy were inversely associated with the risk of autism in children (35–37). Lower first trimester maternal serum levels of 25(OH)D were associated with an increased risk of developing autism in offspring (38). In a study of 4,229 mother-child pairs, Vinkhuyzen et al. found that scores of autism-like behavior (measured by the Social Responsiveness Scale) in 6-year-olds were significantly greater among children whose mothers had VD deficiency in the second and third trimesters (39). Multiple meta-analyses on the association between ASD and VD levels have shown that 25(OH)D levels in children with ASD are significantly lower than those in healthy controls (40), and insufficient VD levels during pregnancy are a risk factor for ASD in offspring (41).

### High-Dose VD Can Improve the Core Symptoms of ASD

Jia et al. administered VD to children with ASD and found that high-dose supplementation with VD could improve the core symptoms of the disorder (32, 42–44). In an international open-label prospective study, mothers who had a child with ASD were given 5,000 IU of daily VD supplementation when they became pregnant again, and the mothers continued to give the newborns 1,000 IU of daily VD supplementation after delivery until the age of 3 years. The prevalence (5%) was significantly lower than the reported comorbidity rate of ASD siblings (20%) (45). In contrast, Aizza and Kerley et al. found that VD was ineffective in improving the clinical symptoms of ASD (46), but the therapeutic dose of VD used in those two studies (2,000 IU/day) was much lower than that of Jia et al. (150,000 IU, muscle, once/month for 3 consecutive months), and the serum 25(OH)D levels of the children with ASD at the end of the study were <40 ng/ml. Another study showed that under the condition of supplementing the same dose of VD (2,000 IU/day), the level of VD in children with ASD was significantly lower than that in children with asthma (47). These findings have been suggested that children with ASD require higher doses of vitamin D supplementation and that increasing the levels of 25(OH)D in the serum of children with ASD to above 40 ng/ml can exert a therapeutic effect (48). The latest randomized controlled trials and meta-analyses of VD in the treatment of ASD have also shown that VD can improve the core symptoms of ASD (49, 50).

### Animal Model Studies Suggest That VD Deficiency Is Associated With ASD

Valproic acid (VPA), a deacetylase inhibitor, is an anticonvulsant and mood-stabilizing drug. Prenatal exposure to VPA can induce ASD behavior in male offspring, which is one of the methods for constructing ASD animal models (51). Studies have shown that the serum 25(OH)D levels of VPA-induced ASD animal models are significantly lower than those of normal controls. Compared with controls, pups of VD-deficient rats (VDD rats) fed a diet deprived of VD exhibit altered ultrasonic vocalizations and stereotyped repetitive behaviors, as well as impaired social interactions during adolescence (52). Early VD supplementation in infant rats with VPA-induced ASD significantly improves related developmental and behavioral issues (53).

In conclusion, VD deficiency is closely related to the pathogenesis of ASD, and supplementation with high-dose VD can improve the core symptoms of ASD. However, the mechanism of interaction between VD and ASD is still unclear.

## The NO Signaling Pathway and ASD

NO is a highly active intracellular and intercellular messenger molecule that is widely distributed in various tissues, especially nervous tissue. NO has physiological functions such as immune regulation, neurotransmission, blood pressure regulation and platelet aggregation inhibition, and changes in its concentrations are closely related to physiological functions (54). In the central nervous system, NO is both a neurotransmitter and a neuromodulator. At low concentrations, NO plays physiological roles in the functions of nerve cells and vascular cells, while at high concentrations, NO can be toxic and lead to cell death, which is related to the pathogeneses of various neurological diseases, including stroke and neurodegenerative diseases (55).

NO is generated from Arg in a reaction catalyzed by NO synthase (NOS). There are two types of NOS: native and inducible. Native NOS is inherent in cells and is divided into neuronal NOS (nNOS) and endothelial NOS (eNOS). Under normal physiological conditions, NO is produced in appropriate amounts through the action of native NOS, thereby exhibiting important physiological functions. nNOS is the major subtype of NOS in the central nervous system. The N-methyl-D-aspartate receptor (NMDAR) at the postsynaptic membrane is activated by glutamate (Glu), which in turn causes Ca^2+^ influx, activates nNOS, catalyzes Arg reactions, and generates citrulline and NO. NO acts on adjacent neurons through diffusion, thereby inducing long-term potentiation (LTP) related to learning and memory and exerting its biological activity (56). eNOS enables endothelial cells to continuously release NO so that vascular smooth muscle is in a state of relaxation, which plays an important role in the regulation of cerebral blood flow. iNOS is mainly distributed in glial cells (including microglia and astrocytes) in the brain. Unlike native NOS (nNOS or eNOS), inducible NOS (iNOS) does not exert biological activity under normal physiological conditions; rather, it is generated and activated only under pathological conditions (such as inflammation, cancer, trauma, etc.) to further generate large amounts of NO.

**Figure 1:**
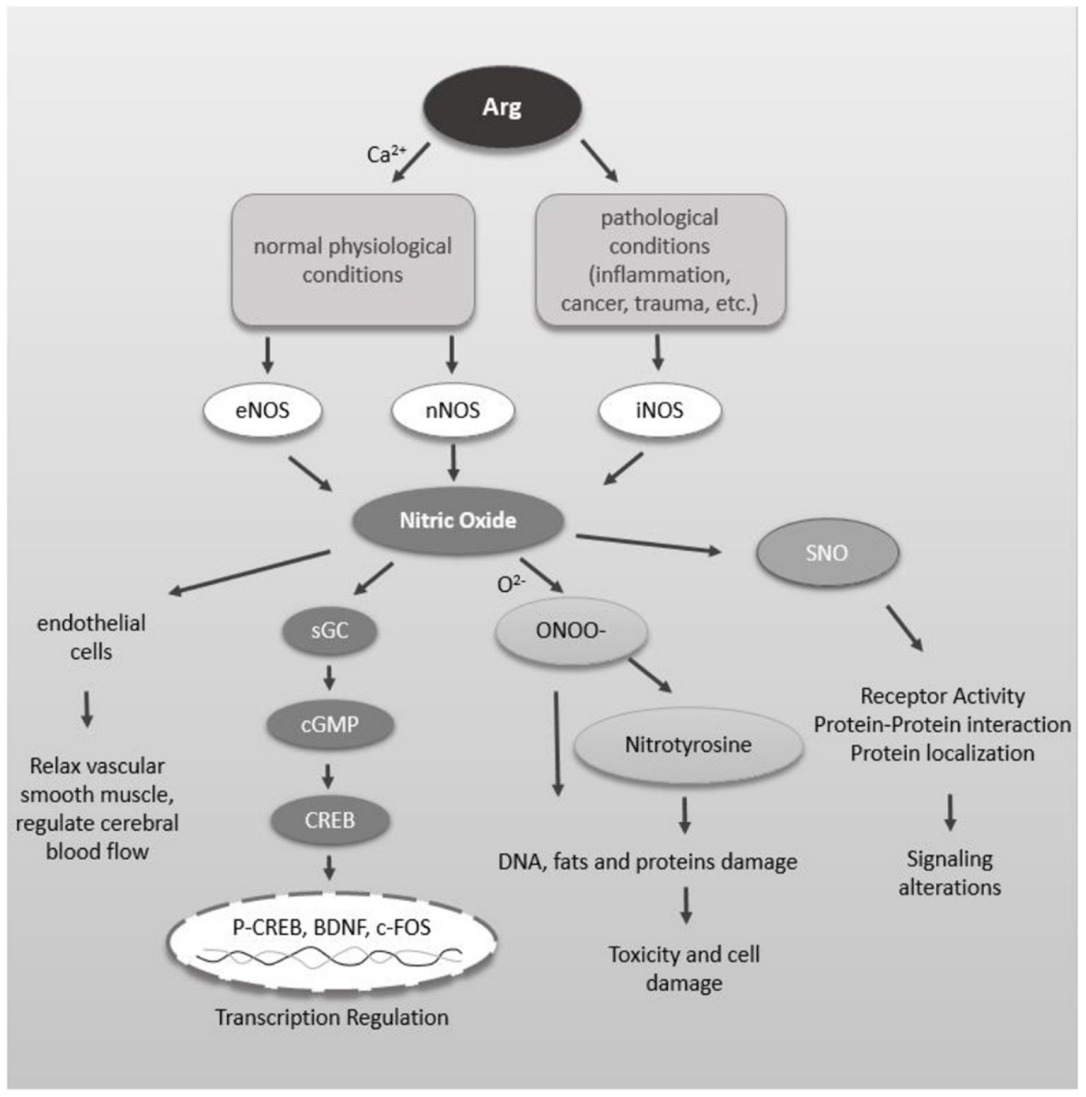
Schematic diagram of NO function in the brain. NO can relax vascular smooth muscle, thereby regulating cerebral blood flow. Arg is catalyzed by different NOS to generate NO under physiological and pathological conditions. NO activates soluble guanylate cyclase to produce cGMP which interacts with many intracellular proteins such as PKG. PKG leads to CREB phosphorylation which leads into transcriptional activation of different genes. NO, directly and indirectly, leads to S-nitrosylation (SNO) of many proteins and receptors. SNO of proteins can alter the receptor activity, protein-protein interaction and protein localization leading to alteration in signaling. Increased level of NO increases proxynitrite formation, tyrosine nitration of proteins, which ultimately may lead into cell death.

There are three main pathways downstream of NO. First, NO can activate soluble guanylate cyclase to generate cGMP, which leads to the transcriptional activation of different genes through the cGMP-PKG-CREB pathway. Second, NO can directly or indirectly lead to S-nitrosation (SNO) of many proteins and receptors, thereby changing the activity of receptors, the interactions between proteins and the localization of proteins, etc., resulting in changes in related signaling pathways (57–60). Dysregulation of NO and SNO signaling is involved in the progression of many neurodevelopmental, neurobehavioral and neurodegenerative diseases. In addition, elevated NO levels increase nitrosative stress, nitrite formation, and tyrosine nitration of proteins and may ultimately induce cell death. NO reacts with superoxide anion radicals (O^2−^) to generate peroxynitrite, which ultimately damages DNA, fats and proteins during oxidative stress (61, 62) (Figure 1).

Although the pathogenesis of ASD is unclear, neurotransmitters, oxidative stress, and immune and nutritional factors are believed to play important roles in the pathogenesis of ASD. These factors are closely related to the NO signaling pathway. Multiple studies have shown that the levels of NO signaling pathway members are altered in children with ASD. These findings suggest that NO may play an important role in the pathogenesis of ASD.

### Children With ASD Have Abnormal Levels of Molecules in the NO Signaling Pathway

Both clinical studies and meta-analyses have found that serum and red blood cells (RBC) NO or nitrite a (metabolite of NO) levels in children with ASD are higher than those in normal children (63–68). Abnormal transport of L-Arg, the raw material of NO, is also related to ASD. Metabolomics studies have found that children with ASD have increased plasma Arg levels and decreased renal Arg clearance (69). Arg is transferred into neurons via cationic amino acid transporter 3 (CAT-3), and genetic testing of children with ASD has identified missense mutations in the *CAT-3* gene (70).

### In Addition, There Is Some Indirect Evidence That ASD Is Closely Related to NO

ASD patients have abnormal levels of neurotransmitters, and abnormal Glu metabolism is considered to be one of the causes of ASD (71). Glu can activate NMDARs, thereby activating nNOS and resulting in NO production. Changes in Glu levels can lead to changes in NO levels in the nervous system. Immune disturbance mechanisms are also recognized to be involved in the pathogenesis of ASD. Lipopolysaccharide (LPS), a complex of lipids and polysaccharides, is the main component of the cell walls of gram-negative bacteria. LPS injection in pregnant rats can cause ASD symptoms in offspring and is thus currently a commonly used method to induce an immune ASD model in rats (72). However, LPS can induce the expression of iNOS in glial cells (73), thereby causing the production of large amounts of NO. In addition, there is an imbalance between oxidation and antioxidant capacity in children with ASD, and oxidative stress can increase the production of NO by neurons, which has a synergistic effect with the neuronal damage caused by oxygen free radicals (74). Furthermore, the ratio of males to females in ASD is ~4–5:1; this sex difference in the incidence of ASD has long been a popular research topic. Notably, NO is closely related to the hypothalamic-gonadal axis, and the activity of nNOS in neurons is also strictly regulated by estrogen (75). Therefore, NO is closely related not only to ASD but also to the intersection of the abovementioned pathogeneses of ASD and is very likely to be a direct target for the treatment of ASD induced by various causes.

## VD and NO

1,25(OH)_2_D is closely related to NO production, and 1, 25(OH)_2_D and NO can modulate each other's concentrations. VD is a steroid hormone that is mainly derived from the conversion of 7-dehydrocholesterol in the skin under the action of ultraviolet rays. After two-step hydroxylation, the active product 1,25(OH)_2_D is formed, which binds to VDR. VDR forms a heterodimer with retinoic acid receptor (RXR), which specifically recognizes the VD response element (VDRE) and regulates the transcription of target genes. The VD signaling pathway can regulate NO and NOS levels. In a study on VDR-knockout mice, the loss of VDR resulted in decreased levels of native NOS and increased expression of arginase 2, which catalyzes the hydrolysis of Arg. Arginase 2 further competed with NOS for Arg to hydrolyze it into ornithine (Orn) and urea (UR), resulting in reduced Arg bioavailability and reduced NO levels (76). *In vitro* studies have found that 1,25(OH)_2_D can inhibit LPS-stimulated changes in iNOS mRNA levels in macrophages and significantly reduce NO production (77). 1,25(OH)_2_D also regulates NO production and iNOS expression in macrophages, endothelial cells, osteoblasts, microglia, macrophages, and astrocytes (78, 79). Population studies have revealed significant negative correlations between serum NO and VD levels in normal adolescents (80). In addition, NO can inversely regulate 1,25(OH)_2_D levels. CYP27B1 is a 1α hydroxylase gene whose expression is bidirectionally regulated by NO levels. The transcription of CYP27B1 can be upregulated at low concentrations of NO but inhibited by high concentrations of NO, thereby further affecting the levels of 1,25(OH)_2_D (81). Therefore, VD levels are closely related to NO levels, and the two influence each other.

## Conclusions

VD deficiency during pregnancy and early brain development is an important environmental risk factor for ASD, and high-dose VD can improve the core symptoms of ASD. The serum VD levels in children with ASD are significantly negatively correlated with the levels of the raw material for NO synthesis, Arg. NO, one of the most important signaling molecules in the brain, is involved in the occurrence of various brain-related diseases and is an important pathological molecule in ASD, Its role in ASD is mediated by a series of mechanisms, such as neurotransmitter imbalance, immune disorders, and oxidative stress. VD can regulate the levels of molecules in the NO signaling pathway. This review hypothesizes a potential mechanism in which abnormal levels of NO during the critical period of brain development caused by various factors (including abnormalities in neurotransmitters, immune disorders, etc.) are important factors in the pathogenesis of ASD, and VD affects the pathogenesis and severity of ASD by regulating NO levels. If this hypothesis is verified, monitoring NO levels during pregnancy and early brain development may become an important strategy for ASD risk prediction, and NO levels may guide the timing and dosing of VD supplementation. Researches on this pathway could contribute to detect more objective criteria for the diagnosis of ASD. The combination of VD and NO- related issues might indicate possible subtype of ASD. Eventually this may lead to more effective prevention and treatment of ASD.

## Author Contributions

BW, LX, and HD contributed to conception of the review and wrote sections of the manuscript. BW, HL, and XY participated in the literature search. BW wrote the first draft of the manuscript. All authors contributed to manuscript revision, read, and approved the submitted version.

## Conflict of Interest

The authors declare that the research was conducted in the absence of any commercial or financial relationships that could be construed as a potential conflict of interest.

## Publisher's Note

All claims expressed in this article are solely those of the authors and do not necessarily represent those of their affiliated organizations, or those of the publisher, the editors and the reviewers. Any product that may be evaluated in this article, or claim that may be made by its manufacturer, is not guaranteed or endorsed by the publisher.
